# Microencapsulating Alginate-Based Polymers for Probiotics Delivery Systems and Their Application

**DOI:** 10.3390/ph15050644

**Published:** 2022-05-23

**Authors:** Xiaochen Wang, Shukun Gao, Shuaiting Yun, Mingjing Zhang, Liyang Peng, Yingxiu Li, Yanxia Zhou

**Affiliations:** Marine College, Shandong University, Weihai 264209, China; 202117736@mail.sdu.edu.cn (X.W.); 201900810266@mail.sdu.edu.cn (S.G.); 202017678@mail.sdu.edu.cn (S.Y.); 202017682@mail.sdu.edu.cn (M.Z.); 202017692@mail.sdu.edu.cn (L.P.); yingxiuli@sdu.edu.cn (Y.L.)

**Keywords:** probiotic, alginate, chemically modified, physically modified, microencapsulate

## Abstract

Probiotics exhibit many health benefits and a great potential for broad applications in pharmaceutical fields, such as prevention and treatment of gastrointestinal tract diseases (irritable bowel syndrome), prevention and therapy of allergies, certain anticancer effects, and immunomodulation. However, their applications are limited by the low viability and metabolic activity of the probiotics during processing, storage, and delivery in the digestive tract. To overcome the mentioned limitations, probiotic delivery systems have attracted much attention. This review focuses on alginate as a preferred polymer and presents recent advances in alginate-based polymers for probiotic delivery systems. We highlight several alginate-based delivery systems containing various types of probiotics and the physical and chemical modifications with chitosan, cellulose, starch, protein, fish gel, and many other materials to enhance their performance, of which the viability and protective mechanisms are discussed. Withal, various challenges in alginate-based polymers for probiotics delivery systems are traced out, and future directions, specifically on the use of nanomaterials as well as prebiotics, are delineated to further facilitate subsequent researchers in selecting more favorable materials and technology for probiotic delivery.

## 1. Introduction

The FAO/WHO definition of probiotics has been widely adopted as: “live microorganisms which when administered in adequate amounts confer a health benefit on the host” [1]. However, a more grammatically correct definition would be “live microorganisms that, when administered in adequate amounts, confer a health benefit on the host” [2]. This definition covers a wide range of microbes and applications, while capturing the essence of probiotics (microbial, viable, and beneficial to health). Probiotics with significant pharmacological activity mainly include *Lactobacillus* species, *Bifidobacterium* species, *Bacillus* species, *Saccharomyces* species, and *Escherichia coli* Nissle 1917 [3,4,5]. Their pharmacological activities have been investigated in animal models, such as the prevention and treatment of diarrheal diseases (acute infantile diarrhea, antibiotic-associated diarrhea, nosocomial infection) [6,7], prevention of systemic infection [8], management of inflammatory bowel disease [7,9], immunomodulation [8], prevention and treatment of allergies [10], anticancer effects [11], treatment of cholesterol, and relief of lactose intolerance [10]. In recent years, probiotics have been extensively studied as a treatment option for various diseases such as obesity [12], diabetes [13], cancer [14], human immunodeficiency virus infection [15], and irritable bowel syndrome [16]

**Figure 1:**
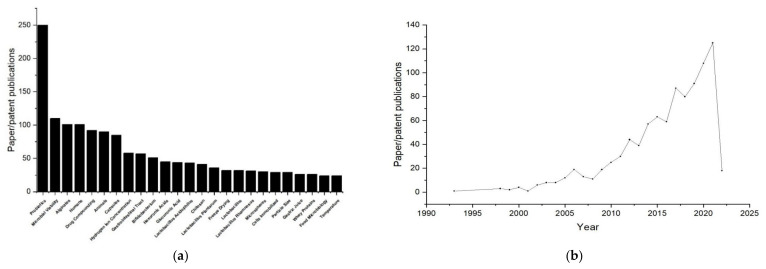
(**a**) Statistical analysis (Based on Mesh Subject Headings) of published papers and patents on probiotics encapsulated within the past years in Web of Science (https://www.webofscience.com/wos/alldb/basic-search, accessed date: 18 March 2022) with the key words of “probiotic and encapsulate”; (**b**) statistical analysis of annual publications of published papers and patents on alginate-related aspects of probiotics within the past years in Web of Science (https://www.webofscience.com/wos/alldb/basic-search, accessed date: 18 March 2022) with the key words of “probiotic and alginate”.

Probiotic delivery systems are critical for ensuring that a sufficient amount of probiotics reach the large intestine and are released in the colon [17], since free probiotics are prone to be easily destroyed by the harsh conditions within the human upper gastrointestinal tract (GIT), such as the presence of antimicrobial lysozyme in the mouth [18], the low pH conditions in the stomach [19], the bile salts and digestive enzymes in the small intestine [20], and other complex factors including osmotic pressure and oxidative stress through the gastrointestinal tract. Microencapsulation is a widely applied technique for probiotic delivery system, which can package various bioactive components in protective shells that provide physical barriers to improve the viability and bioavailability of probiotics [6,21,22,23].The probiotic delivery carriers of microencapsulated shells, which have been reported in the past, are mainly natural polymers [17,24] such as k-carrageenan, alginate, pectin and starch derivatives, gum arabic, gellan, xanthan, and animal proteins [25,26]. Among these probiotic delivery carriers, alginate (Alg) has attracted much attention due to its excellent physicochemical and mechanical properties [27,28,29], including simple structure, simple raw materials, low toxicity, mild processing, and ease in forming a gel matrix around the bacteria. Analysis of the data in recent years shows that the number of alginate-related studies published ranks third in terms of probiotic encapsulation (Figure 1a). Related publications have increased, especially in the past 10 years (Figure 1b). From its vast use in food processing and biotechnology in a constantly growing market of approximately USD 10 billion by 2021, the use of alginate’s attractive properties has expanded to the biomedical and pharmaceutical industries [30]. Paul de Vos et al. mentioned that the most commonly used polymer in encapsulation studies is alginate, which has been well-characterized [31]. The composition and sequence characteristics of alginate have been intensively studied [32]. It can be said that the cellular encapsulation material with the highest chance of success for clinical applications is alginate, as it is the best polymer to document and study [31]. To improve the properties of alginate, physical and chemical modifications have been recently developed for alginate-based microencapsulation of probiotic cells in combination with other materials including chitosan, zein, gum arabic, cellulose, starch, whey protein, gelatin, and pectin [6]. In this review, the alginate-based delivery systems loading various types of probiotics that achieve good performance for various diseases are summarized. Furthermore, this paper will classify the alginates used to encapsulate probiotics in a physical as well as chemically modified manner, which is rarely used. In addition, the viability and protective mechanisms of alginate-based probiotic delivery systems are discussed. Finally, the prospects and challenges of probiotic delivery systems are pointed out.

## 2. Alginate-Based Probiotics Delivery

### 2.1. Natural Alginate

**Figure 2:**
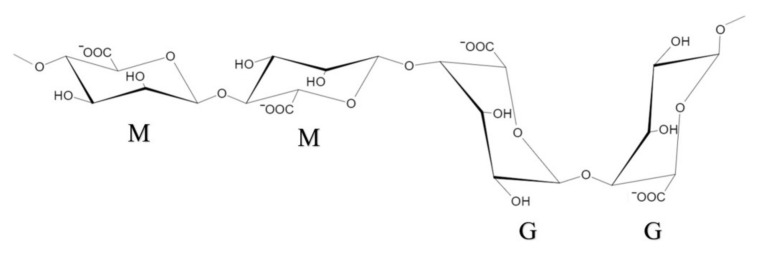
Structure of α−l-guluronic and β−d-mannuronic alginate residues.

**Figure 3:**
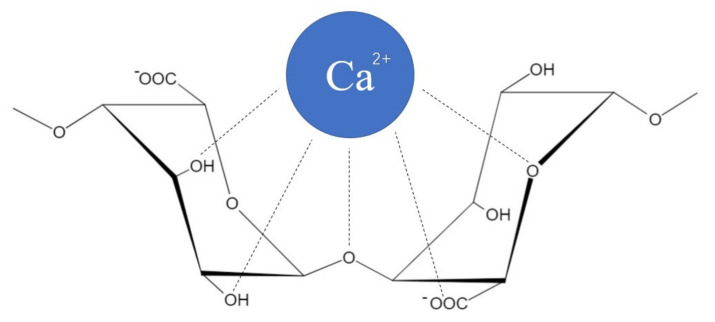
Interaction between divalent cation and G monomers of alginate in the “egg−box model”.

Alg is a linear hydrophilic polysaccharide derived from brown algae, composed of two monosaccharide units: 1–4 linked β−d−mannuronic (M) and α−l−guluronic (G) acids (Figure 2). Hydrogel formed by Alg is indissoluble in acidic conditions, which may protect probiotics from gastric acid and bile [3,33]. To prepare microcapsules, alginate can form hydrogels by the cooperative binding of divalent cations and the dimerization of G residues [34,35,36]. The gelation mechanism of alginate is often referred to as the “egg-box” model [6,36], in which the binding of G chains on opposite sides forms a diamond-shaped hole containing a hydrophilic cavity that binds the Ca^2+^ using the oxygen atoms from the carboxyl groups by multi-coordination (Figure 3). This configuration results in egg-box junction zone shape, and it can be achieved by using other types of divalent cations [34,35]. The ratio and sequence of G and M residues affect the physical properties of alginate hydrogels. Alginates with high G content will form stiffer and more porous gels and maintain their integrity for a longer time. On the contrary, alginates with high M content can form softer and less porous gels, which easily disintegrate over time [37,38]. Given that the gel formation is processed under mild conditions by ion crosslinking at room temperature and the good permeability of alginate gels makes it easy to exchange air and nutrients and release metabolites, alginate has become a popular support for probiotic delivery [39].

Numerous research studies have focused on the Alg delivery of probiotics, which confirmed the advantages and feasibility of alginate as a probiotic delivery carrier. Table 1 summarizes research studies on encapsulation of probiotics with Alg undertaken recently. Alg can be an excellent carrier for delivering probiotics, with high encapsulation efficiency and a significant increase in the survival rate of probiotics. An overview of some articles will thus be given.

Microencapsulation of probiotics has received much attention in recent years. Yeast, one of the common probiotics, has also been experimented on for immobilization and can be used for continuous fermentation [25,43,44]. Yeast microencapsulated with alginate can survive for a long time at low temperatures and improves the survival rate of probiotics [45]. In addition, it was found that yeast in alginate microcapsules showed no significant change in strain concentration before and after gastrointestinal exposure, indicating that alginate microcapsules are a suitable vehicle for releasing probiotics in the intestine [40,45,46]. 

Alternatively, sodium alginate is a polysaccharide commonly used to formulate edible coatings because of its biodegradability, biocompatibility, low toxicity, and film-forming properties [47], which exhibit great value in storage. A recent study showed that loading potential probiotic fruit-derived lactic acid bacteria (LAB) strains into sodium alginate (SA) coating could effectively reduce the anthracnose lesion development in guava and mango contaminated with either of the tested *Colletotrichum* strains during storage [41], which can significantly improve the overall postharvest quality and long storage resistance of fruits [41]. 

Alginate microcapsules also have relevant applications in aquaculture. Probiotics such as *Bacillus licheniformis* and *Lactobacillus rhamnosus* can positively affect shrimp and other aquatic species by acting as growth promoters, enhancing immune responses, and improving water quality throughout by altering the presence of other microorganisms in the water and soil [48,49]. Vega-Carranza et al. encapsulated the marine probiotic *Bacillus licheniformis* in alginate particles (AMPs) by ionic gelation, which significantly improved the probiotics’ storage stability and seemed to be suitable for targeted delivery of these probiotic bacteria into the intestine of shrimp [42]. The targeted release of *B. licheniformis* in the intestine may be due to the modulatory effect of the wall material (alginate) on certain endogenous enzymes of the shrimp hepatopancreas. Some probiotic compounds (e.g., alginate or maltodextrin) can induce the production of endogenous enzymes (e.g., amylase) by the hepatopancreas, which are then excreted into the intestine through digestion. These enzymes hydrolyze the glycoside bonds of polysaccharides, thus allowing the probiotic bacteria to be released directly into the intestinal tract [50]. It is worth noting that alginate microcapsules not only have advantages in improving the stability, survival rate, and targeting of probiotics, but also have simple, fast, and cheap production, which has great potential for application in mariculture [42].

Alginate is an excellent probiotic carrier to improve the survival rate of probiotics; however, it has also been reported that alginate alone has some disadvantages for encapsulating probiotics. Some studies mention that alginate microbeads protect probiotics during storage, but do not protect probiotics well in low pH conditions (e.g., in gastrointestinal fluids) compared to microcapsules containing a coating (e.g., alginate-probiotic microbeads coated with chitosan) [51,52]. Hansen et al. found that the porosity of alginate gel increases with the presence of some bacteria and changes with the concentration of H^+^, which leads to the limitation of probiotic protection [53,54]. Razavi et al. also found that high porosity of alginate microbeads leads to limitations such as rapid release of loaded molecules, low probiotic encapsulation efficiency, easy degradation in acidic environment, and poor transport of probiotics to the intestine [6]. Therefore, chemical or physical modifications of alginate are needed to improve the above disadvantages.

### 2.2. Chemically Modified Alginate

Alginate is a linear anionic polysaccharide, so substances with opposite charges can be attracted to each other with fucoidan to form a complex gel to co-embed probiotics. Commonly used polycations are natural polymers (e.g., chitosan), proteins (e.g., whey protein), and synthetic polymers (e.g., polyvinyl alcohol (PVA)) (Table 2). The table shows that chitosan and protein are two common materials used to chemically modify alginate. Microencapsulation of probiotics with chemically modified alginate not only improves the encapsulation efficiency of probiotics, but some of them also have pH responsiveness, which enables probiotics to act better in the intestine.

#### 2.2.1. Chitosan

Chitosan is a cationic polysaccharide with positive charge and can form an electrolyte composite gel with anionic sodium alginate. Alginate (AG)/chitosan (CS) composite scaffolds are widely applied in biomedical fields (e.g., hemostasis, wound healing, and tissue engineering) because they possess excellent biocompatibility, biodegradability, mechanical strength, and antibacterial properties, and they can activate blood coagulation and absorb wound exudate [28,70].

Recent studies have found dysregulated interactions among intestinal bacteria, the intestinal barrier, and the gut-associated immune system in patients with inflammatory bowel disease (IBD) [71,72], and oral probiotics show considerable potential as an alternative drug for the treatment of IBD [73]. *Ligilactobacillus salivarius* Li01, isolated from healthy individuals, can protect the intestinal barrier, reduce serum inflammatory cytokine levels and bacterial translocations, and increase the abundance of the gut microbiota [74], implying that strain Li01 has great potential in preventing or reducing colonic inflammation. However, strain Li01 is highly susceptible to environmental factors such as oxygen, gastric acid, and bile salts [75]. Yao et al. designed a delivery system which encapsulated a single Li01 cell layer-by-layer (LbL), using chitosan and alginate to protect probiotics [55]. The encapsulation process begins with the coating of a chitosan (positively charged) layer followed by an alginate (negatively charged) layer to form a bilayer. It was found that the swelling properties of CA bilayers in SIF were stronger than in SGF. This may be attributed to the protonation and deprotonation of alginate carboxylic acids under different pH conditions. In SGF, the carboxylic acids undergo pronation and therefore the swelling of cross-linked alginate is very limited. In contrast, under neutral conditions, deprotonation of the carboxylic acid occurs, resulting in a larger swelling of the alginate gel [76,77]. Due to the good swelling properties of CA, the bilayer may promote the penetration of stimulators and provide good protection to the cells. In addition, the LbL delivery system can enhance mucoadhesive properties. The combination of chitosan, which has strong adhesion to mucin but slightly poor biocompatibility, with alginate, which has high mucosal biocompatibility as well as moderate adhesion [78], can improve the performance of the carrier. Sodium alginate and chitosan are mucoadhesive and can adhere to mucus to prolong retention time, thus promoting probiotic colonization in the intestine [56]. After the probiotics are covered by the bilayer, the mannose content in alginate is not involved in the formation of the ionic gel network and plays a key role in adhesion through the formation of hydrogen bonds and other van der Waals interactions [79]. It was found that the longer the contact time, the stronger it is. These added functions for Li01 cells help them to improve DSS-induced colitis in mice [55].

**Figure 4:**
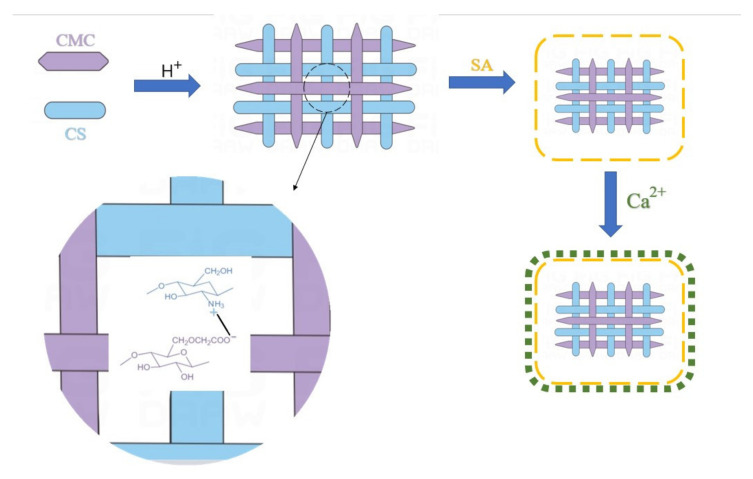
Formation diagram of the reticulated shell structure of hydrogel beads.

To enable probiotics to reach the gastrointestinal tract accurately, pH-responsive hydrogels are considered to be an ideal vehicle for oral probiotics because they exist stably in the strong acidic environment of the stomach but unstably in the weak acidic environment of the intestine [80]. Given the requirements for biocompatibility, biodegradability, and nontoxicity of drug vectors, many biomacromolecules have been applied to manufacture pH-responsive hydrogels [81,82]. It is shown that sodium alginate (SA) and carboxymethyl cellulose (CMC) are linear polysaccharides containing a large number of carboxylate groups and can be non-covalent cross-linked with the cationic polymer chitosan (CS) to construct water-filled 3D networks with pH response behavior [83,84]. Furthermore, several studies have shown contradictions between the pH sensitivity of the hydrogel and its structural stability (including swelling, thermal stability, and network robustness [85]). For example, the calcium alginate (CA)-CMC microbeads prepared by Agarwal et al. showed pH-dependent swelling and prolonged sustained release, but their sparse internal network was not stable [86]. Therefore, it is a huge challenge to synthesize hydrogels with structural stability and pH sensitivity. Wang et al. designed a new strategy for constructing the reticulated shell structure based on two systems of CMC/CS and SA/Ca^2+^, namely, pH-sensitive CMC/CS/SA hydrogel beads with the reticulated shell structure (Figure 4), for delivering *Bacillus subtilis* natto to the intestinal tract. The electrostatic interaction between CS and SA was found to be stronger than the hydrogen-bonding interaction between CMC and SA. It is further speculated that the complete mixing of CS and CMC before the introduction of SA can both make full use of electrostatic effects to occupy the protonation amine group of CS and retain the abundant SA carboxy group to chelate with Ca^2+^. A reticulated shell structure composed of a strong outer shell and inner porous network was formed in the experiment. The structure exhibited many prominent behaviors including obvious pH sensitivity, high thermal stability, high probiotics load, desired sustained release, and high storage stability [61]. 

#### 2.2.2. Protein

Previous studies have shown that the inclusion of proteins and peptides in the encapsulation of probiotics contributes to the mechanical properties of the beads, fermentation activity, acid and bile tolerance, and the survival of probiotics under simulated gastrointestinal conditions, as well as improving biological activity and antioxidant properties [87,88]. Whey proteins and peptides are not only rich in nutritional value but also have excellent gelation properties, so they are used as probiotic carriers [63]. Divalent cations, such as Ca^2+^, can induce protein aggregation in different ways [89]. Whey proteins alone mechanically build insufficiently strong and stable matrices without the addition of any additives. Luckily, the combination of whey proteins and alginate can solve the disadvantage above and tighten them together in a rigid structure, which has received widespread attention.

A mix of *Streptococcus thermophiles* and probiotics trains *Lactobacillus delbrueckii* sp. *bulgaricus*, *Lactobacillus acidophilus*, and *Bifidobacterium bifidum* encapsulated in two different carriers (whey protein-alginate and whey protein hydrolysate-alginate) to investigate fermentative activity of encapsulated cultures used for fermentation of whey-based substrate, effect of fermentation on carrier properties, and viability of probiotic strains during simulated digestion. The results showed that the addition of whey protein concentrate (WPC) and whey protein hydrolysate (WPH) to an alginate carrier improved the mechanical properties of the beads and enhanced the antioxidant capacity of the beverage [63]. It was found that under the isoelectric point, positively charged proteins interact with negatively charged alginate, while above the isoelectric point, the presence of Ca^2+^ induces negatively charged protein gels, similar to alginate hydrogels [90]. Due to the different nature of proteins and the possibility of cross-linking with sodium alginate at different pH values, it can be concluded that alginate-whey protein complex is pH-sensitive. Interestingly, the results of the study found that the microbeads did not shrink during fermentation but increased in diameter. This is different from the previous finding that calcium alginate microbeads shrink and increase in stiffness when pH is reduced. It can be clearly seen that the addition of WPC and WPH to alginate helps to improve the mechanical properties of the beads before fermentation and leads to a greater increase in mechanical strength during fermentation [63]. In addition, the higher porosity of the protein-alginate matrix allows for better material exchange between cells and matrix, resulting in a substantial increase in the number of surviving cells, in agreement with other literature [63,91]. The addition of WPC and WPH improved the viability of probiotic bacteria under simulated gastrointestinal conditions. Interestingly, the combination of WPC with alginate provides a very porous matrix, while the shorter peptide chain, WPH, provides a less porous carrier, the latter providing better cellular protection against adverse external factors [63]. 

In addition, whey protein isolate (WPI) has been used as a carrier for immobilized probiotics [66]. Dehkordi et al. found a direct relationship between the encapsulation efficiency of probiotics and the polymer concentration [66]. The variation in encapsulation efficiency could be mainly due to the high concentration of polymers, which increases ionic cross-linking and forms denser membrane by repeating the opposite charge group. The effect of denaturing whey isolated proteins in combination with sodium alginate on probiotic survivability had been studied and compared with whey isolated proteins, showing that denaturing whey proteins has a better protective effect than whey isolated proteins [65]. This may be due to the formation of the unfolded polypeptide parts, which allows for protein-protein interactions, hydrogen binding, and disulfide crosslinking. 

In addition to whey protein, other proteins can be used to immobilize probiotics, such as caseinate, a casein derivative that was first used as a material for encapsulating probiotic strains in 2009. Li et al. used transglutaminase (TGZ) to induce casein gelation to form casein microcapsules (CM), which achieved co-embedding of *Lactobacillus paracasei* and lactitol, and coated the surface of casein microcapsules with sodium alginate (AM) [92]. It was found that there was agglomeration between microcapsules after alginate encapsulation, leading to an increase in particle size. The size of microcapsules has an important impact on the viability of probiotics and organoleptic characteristics. In general, larger microcapsules provide better protection for probiotics, but may negatively affect the sensory function of the product [93]. Alginate-encapsulated casein microcapsules improved the survival of *L. paracasei* under simulated gastrointestinal conditions. This may be due to the formation of stable aggregates between the positively charged sodium caseinate and sodium alginate through electrostatic interactions, thus reducing the contact between sodium caseinate and pepsin and improving the survival of probiotic bacteria [94]. Microencapsulation of probiotics by combining alginate and protein allowed the preparation of microspheres with stable morphology and suitable size for food-grade drug delivery systems, which have high application value [92].

#### 2.2.3. Wild Sage Seed Mucilage (WSSM)

Prebiotics are defined as “selectively fermented ingredients that allow specific changes, both in the composition and/or activity of the gastrointestinal microflora, and confer benefits upon host well-being and health” [95]. The addition of prebiotics to the structure of Alg microcapsules can increase the activity of encapsulated probiotics [96,97]. Wild sage seed mucilage (WSSM) is a galactomannan with a rigid rod-like conformation and is presumed to be a good prebiotic with great potential as a stabilizer, thickener, or emulsifier in food products [98]. *Lactobacillus casei* were immobilized with a mixture of sodium alginate and WSSM and their properties were investigated [67]. DSC of the samples showed that the addition of WSSM increased the melting temperature (Tm), glass transition temperature (Tg), and melting enthalpy (ΔH) of the microcapsules. This suggests that the application of WSSM in the microcapsule structure of Alg can improve the integrity and stability of the microcapsules and effectively protect probiotics from the harmful gastrointestinal environment [67]. In the current study, the model used to assess the resistance of probiotics to harsh environmental conditions did not include digestive enzymes, which may also inactivate probiotics. The article suggests that future studies should investigate the effects of oral, gastric, or pancreatic enzymes on the survival of free and encapsulated probiotics, and that the stability of encapsulated probiotics during actual food processing and storage is an area that needs further study [67].

### 2.3. Physically Modified Alginate

In addition to the chemical interactions described above, there are also substances bound to alginate by mechanical physical filling or intermolecular hydrogen bonding, such as starch. Table 3 summarizes articles on the microencapsulation of probiotics by physically modified alginate in recent years. Microencapsulation of probiotics by physically modified alginate is beneficial for reduction of carrier porosity, improvement of the survival rate of probiotics, and increase of the storage stability of probiotics. An overview of some articles will thus be given.

#### 2.3.1. Starch

Starch is considered a good candidate for mixing with alginate because it is cheap, abundant, biodegradable, and comes from renewable sources [110]. Moreover, the addition of starch can increase the mechanical strength of the alginate beads and improve bacterial survival during bead making, drying, and storage [111]. Khlibsuwan et al. found that gelatinized starch yielded higher encapsulation efficiency than ungelatinized native starch [112]. To address the disadvantages of alginate microbeads, such as low mechanical strength, microbead shrinkage, poor appearance, and insufficient cell protection, the alginate hydrogels were prepared by mixing alginate with cassava starch [99]. The result showed that the encapsulation efficiency increased linearly with the increasing cassava starch content, which is similar to previous reports crediting starch addition with increasing encapsulation efficiency [100,112]. The even distribution of starch in the beads produces homogeneous beads with a smooth surface and was responsible for the sphericity of beads after drying because the starch filled all the voids inside the beads [101,113]. Alternatively, the introduction of either gelatinized or ungelatinized starch into an alginate dispersion increased the viscosity of the solution. This is the result of the hydroxyl interaction of the starch molecule with the carboxyl group of the alginate through intermolecular hydrogen bonding. Such interaction produces a viscosity synergistic effect in the alginate/starch mixture, thereby increasing the complexity of the matrix network [99,112].

The introduction of electrostatic spinning technology into alginate-starch immobilized probiotic systems revealed that nanocapsules using electrospinning technology significantly improved the acid resistance and survival of the tested bacteria [102]. Although alginate loses its mechanical integrity and bacterial protection in acidic environments, the combination of alginate and starch enhances the structural properties of microcapsules [68]. The molecular structure formed by sodium alginate effectively protects the encapsulated bacteria and improves their survival protection against adverse environmental factors, such as low or high Ph [58,109]. Atraki et al. used electrospinning technique to nano-embed human-intestinal-derived probiotics *Lactobacillus* and *Bifidobacterium* in starch and sodium alginate to improve their survival and viability in a simulated gastrointestinal model [102]. In another study, lactic acid bacteria and *Bifidobacteria* were nanocapsulated with starch and alginate sodium using the electrospinning method to improve their survival and viability in yogurt [103]. The survival rate of the probiotics prepared in the study was higher than previous work, reflecting the high efficiency of the electrospinning method and the good compatibility of starch with sodium alginate, which protected probiotic cells from the adverse effects of gastric acid and bile salts [102]. Notably, this study produced nanocapsules with a higher protective effect compared to nanoemulsification, microemulsification, and extrusion techniques applied in previous studies [59,102,114]. Ramirez et al. reported that alginate and starch granules act as a protective agent against each other and can prevent or reduce the action of digestive enzymes on the inclusions [115]. Alternatively, the experimental results identified electrospinning methods with higher efficiency and function, as well as the biocompatibility of the corn starch and sodium alginate biopolymers in improving the survival of the given probiotics [116]. 

#### 2.3.2. Poly (Vinyl Alcohol)

Recently, nanoencapsulation has been introduced as a technique which can greatly increase bioavailability, enhance encapsulation efficiency, and improve controlled or targeted release due to its larger particle surface area than microencapsulation [117]. In previous studies, different types of biopolymers, such as poly (ethylene oxide) nanofibers, chitosan, and agricultural waste nanofibers, have been used for nanoencapsulation of probiotics. Among the multiple methods for nanoencapsulation of probiotics, electrospinning is recognized as an efficient technique [118,119]. Higher solubility, strength, and use of nontoxic materials are important for the success of the electrospinning process. Thus, materials with the above properties such as poly(vinyl alcohol) (PVA) and NaAlg can be used in the electrospinning process [120]. Among *Lactobacilli*, *L. rhamnosus GG* is a well-known probiotic strain that has several positive roles in human health, especially for allergic diseases [121]. Moreover, these bacteria play an important role in extending the shelf life of some food products [122]. In a more recent study, poly (vinyl alcohol) and sodium-alginate-based nanofibers (VS) were used as a nanocarrier and probiotic bacteria were loaded into VS [108]. In previous studies, most studies used a single type of material to obtain nanofibers [123,124]. In this study, probiotic-loaded nanofibers (<580 nm) were successfully obtained and showed significant inhibition of the growth of certain harmful bacteria, such as the mesophilic aerobic bacteria and the psychrophilic bacteria in the fish fillets, although the average diameter of the nanofibers was increased using three different materials (probiotics, polyvinyl alcohol, and sodium alginate) [108]. Interestingly, alginate combined with electrospinning technology showed excellent results not only on polyvinyl alcohol materials, but also on materials such as polystyrene and starch, which will be described later in detail.

#### 2.3.3. Polystyrene

**Figure 5:**
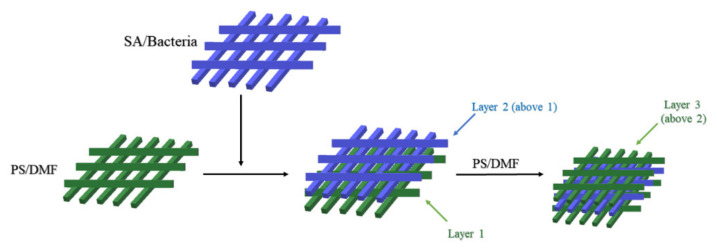
Production of electrospun fibers with encapsulated bacteria.

Grzywaczyk et al. prepared alginate hydrogel beads with immobilized probiotic cells and then the alginate hydrogel beads were sandwiched between two polystyrene (PS) mats via the electrospinning process (Figure 5) [107]. This layer-by-layer microencapsulation process was used to improve the stability and chemical and thermal resistance of the probiotic bacteria as well as to reduce the leaching of the probiotic bacteria. In addition, to improve the thermal stability of the cells, various microencapsulation techniques were used to immobilize probiotics, and their viability and UV resistance were measured [107]. The results showed that the metabolic activity of the cells was reduced by exposure to high temperature and UV irradiation for 24 h, regardless of the preservation technique used. However, electrospun fiber immobilized cells had the highest metabolic activity in the samples analyzed, demonstrating the good protective properties of the produced fibers [107]. Moreover, the good wettability makes the hybrid material a promising tool for industrial applications. This method is mainly applicable to Gram-positive bacteria with cytoarchitecture similar to that of *Lactobacillus* cells [107]. It is noted that the relevant review does not mention more extensive data on polystyrene, and therefore this study can be considered to be a valuable contribution to the current state of knowledge as a proof of usability of the PS nanofibers with alginate hydrogels for microencapsulation of bacteria [107,125].

#### 2.3.4. Carboxymethylpachymaran

**Figure 6:**
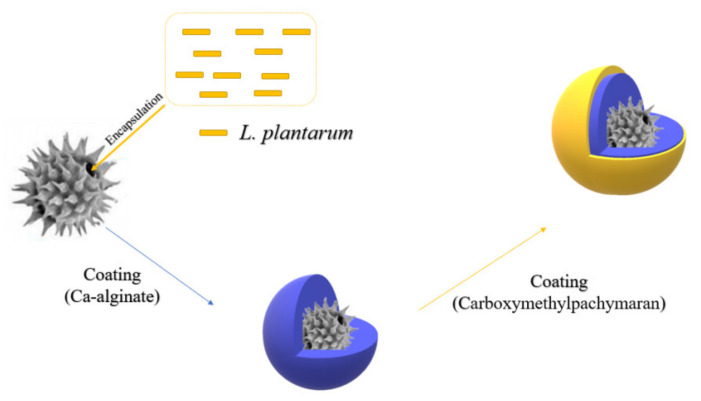
Schematic diagram of a new core-shell structure.

As mentioned earlier, in most cases, the beneficial effects largely depend on the delivery of enough living probiotic cells into the large intestine [126]. However, the activity of probiotics in commercial products is susceptible to processing conditions such as freeze-drying and storage [127,128]. In addition, probiotics always face severe conditions after being ingested in the human gastrointestinal tract (GIT), such as the presence of proteases and unfavorable pH in GIT, which may lead to a substantial decrease in their viability [69,129]. At present, sporopollenin exine capsules (SECs) derived from natural pollen are considered to be a natural microcapsule for the protection of sensitive biomolecules because of their large inner lumen, temperature, pH, ionic strength, and mucosal adhesion resistance [130,131]. However, the presence of many pores on the surface of SEC acts as a double-edged sword, creating access for probiotic encapsulation, but also providing access to digestive juices in GIT, thus reducing the viability of probiotics [132]. Previous studies have shown that carboxymethylpachymaran (CMP) gels have high pH sensitivity in the gastrointestinal tract [133]. More importantly, CMP can mitigate cell damage during freeze-drying, suggesting that CMP may be a novel cryoprotective agent [132]. Therefore, Deng et al. developed a novel core-shell structure with SECS as the core and Ca-Alg/CMP gel as the shell to protect probiotics, aiming to improve the storage and lyophilization stability of probiotics and to achieve their sustained release in GIT for delivery to the human colon after processing and storage in commercial products (Figure 6) [105]. It was shown that in the Ca-Alg/CMP shell layer, the CMP content could influence the swelling behavior and microstructure of the shell layer by affecting the hydrogen bonding between CMP and Alg, thus affecting the release behavior of probiotics. Meanwhile, the introduction of CMP can improve the thermal stability of the shell, which is of great significance for the practical application of this shell in industry [105].

## 3. Applications, Prospects, and Challenges

### 3.1. Applications of Microencapsulated Probiotics

Microencapsulated probiotics are widely used in biomedicine. As mentioned earlier, probiotics have a variety of pharmacological activities and the introduction of probiotics into the gastrointestinal tract may be a potential strategy to restore the balance of the intestinal ecosystem and to prevent or treat disease. Microencapsulated probiotics are well able to deliver probiotics to the intestine and exert therapeutic effects. For example, microencapsulation of Li01 may increase the potential of this probiotic for clinical application in the treatment of IBD [55]. There is experimental evidence that microencapsulation of probiotics into alginate-polylysine-alginate (APA) microcapsules can be used for the treatment of metabolic syndrome [134]. The CS and Alg complex microcapsules of probiotics not only ensure the viability of probiotics, but also improve the adhesion of probiotics to the intestinal tract, which helps probiotics to attach to the target area and grow [56,135]. Microencapsulation can expand the application of probiotics, even for in vitro therapeutic effects. For example, microencapsulated probiotics have shown potential for use in stomatology [136]. In the treatment of periodontal disease, the survival rate of probiotics coated with AlG and CS is improved and has good value-added ability. Among them, CS as coating material increases the specific surface area and adhesion of microcapsules. The coating can also help the sustained release of probiotics [137]. In addition, microencapsulated probiotics can be used for skin inflammation treatment as well as bacterial vaginosis. For example, calcium alginate membrane containing *L. plantarum* has the potential to prevent burn infections [138].

Probiotics also have applications in the field of food. For example, probiotics are widely used in fermented dairy products, including yogurt, cheese, and ice cream [139]. Microencapsulation can improve the viability as well as the biological activity of probiotics during processing, transportation, and storage. Functional beverages with active probiotics culture activity and high antioxidant capacity can be produced by alginate microencapsulated probiotics [64]. Bakery products are an emerging category in the probiotic food sector and are attracting increasing research interest [140]. The addition of alginate and chitosan to microcapsules can effectively protect *Lactobacillus acidophilus* and is an effective method for the production of probiotic bread [140]. Alginate/fish gelatin capsules improve the survival of probiotics during baking and storage and are an effective bread enhancer [106]. In addition, the probiotics can be made into edible films, reducing the loss of viability of *Lactobacillus rhamnosus* during baking, drying, and storage [141].

### 3.2. Prospects and Challenges

Although many studies have successfully immobilized probiotics with alginate and have achieved good experimental results, we still face many challenges. For example, most articles did not explain the therapeutic effect, pharmacological action, and practical application of the probiotics studied. This prevents readers from better understanding the importance of experiments and conducting further research.

It was found that the structural organization of microgels is usually characterized using optical or electron microscopy during research, and it was suggested that other methods can be used more often to detect the structure and distribution of probiotics in microgels, such as atomic force microscopy (AFM) [142] and fluorescein labeling method [24]. In addition, for evaluating the survival and tolerance of probiotic bacteria in delivery systems during gastrointestinal transit, most static in vitro digestion models are used, i.e., using a constant ratio of food to digestive fluid and a constant pH at each digestion step. To better simulate gastrointestinal conditions, dynamic in vitro digestion models can be used [143], such as dynamic gastrointestinal simulator (SIMGI) [144]. In addition, in vitro models are more practical for rapid screening of many different formulations, but they cannot accurately simulate the human intestinal tract. Therefore, more accurate in vivo models could be tested in terms of probiotic delivery systems, which would be the next step forward for most promising novel delivery systems [24]. Notably, some biomaterial-based microencapsulated probiotics are intended for use in humans, which means that the safety and suitability of these encapsulation materials and methods must be evaluated [3].

The application of various novel probiotic encapsulation techniques (e.g., electrostatic spinning technology) has given alginate a broader prospect for microencapsulation of probiotics. The pH-responsive alginate microparticles provide an opportunity for the design of targeted oral colon delivery systems. In addition, the proposal of prebiotics and the co-encapsulation of probiotics and prebiotics is a promising approach to enhance the survival and functional activity of probiotics. However, due to the increasing demand for these components, researchers must strive to find more new sources of prebiotics [67,145]. In addition, the use of nanomaterials may make the microencapsulation and transmission of probiotics more effective [117,146]. The development of alginate hybrid nanocarriers could help to improve the activity, stability, and bioavailability of probiotics and enable the release of probiotic cells at the desired site of action. There have been some relevant studies; however, it is still an area that needs to be further explored. Despite advantages of probiotic delivery systems, the microencapsulation process increases the cost of the final product, which is the limitation for industrial production of probiotics [6]. In the future, more research is needed to test novel strategies for alginate delivery of probiotics to enable their widespread use at the industrial level.

## 4. Conclusions

In this paper, we review the progress of alginate-based polymers for probiotic delivery systems and their applications in different diseases. Probiotics have a wide range of significant pharmacological activities, but low viability and metabolic activity limit their pharmaceutical applications. The alginate-based delivery systems are favorable approaches for factors such as better activity and selectivity, broader pH range, thermal stability, and long-term stability which can enhance the bioactivity in the intestine for applications. Compared with unmodified alginate, modified alginate has many advantages as an ideal polymer for probiotic encapsulation, such as high encapsulation rate, good adhesion, high pH responsiveness for controlled and targeted release, and versatility, which can expand the application of probiotics in various diseases. In addition, this paper identifies the prospects and challenges of probiotic delivery systems. It can be foreseen that with an improved understanding of the health benefits of probiotics and improvements in probiotic delivery technologies, there will be an increasing market for encapsulated probiotics in the future.

## Figures and Tables

**Table 1:** Microencapsulation of probiotics with Alg.

Encapsulated Strain	Application	Remarks	References
*Saccharomyces cerevisiae* strains	--	Encapsulation yield was at least 60% (100% for some strains) and yeasts survived in beads for 30 days at 4 °C.	[25]
*Saccharomyces boulardii* and *Enterococcus faecium*	--	Higher survival rate of *S. burrici* and *E. faecium* (increased by 25% and 40%) at high temperature and high humidity. Higher survival rate in the SGF ^1^ (increased by 60% and 25%) and SIF ^2^ (increased by 15% and 20%).	[40]
Fruit-derived lactic acid bacteria (LAB)	Reduce the anthracnose lesion development in guava and mango.	Sodium alginate coatings loaded with laboratory strains had higher ALDR% (anthracnose lesion diameter reduction) values in guava and mango.	[41]
*Bacillus licheniformis*	--	Cell survival rate for 30 days at 4 °C was 55.58 ± 2.35%. Higher survival rate of alginate-encapsulated bacteria (100.0 ± 7.72%, 62.71 ± 4.81%, 80.79 ± 7.40%, and 51.29 ± 0.42%, respectively) in the simulated shrimp digestive tract (stomach, hepatopancreas, and midgut or hindgut).	[42]

^1^ SGF is short for “simulated gastric fluid”. ^2^ SIF is short for “simulated intestinal fluid”.

**Table 2:** Microencapsulation of probiotics with chemically modified alginate.

**Coating** **Material**	**Encapsulated Strain**	**Application**	**Remarks**	**References**
Alg + CS	*Ligilactobacillus* *salivarius* Li01 (Li01)	Treatment of inflammatory bowel disease.	After 2 h incubation in the digestive solution, the cell number of probiotics remained above 6 log CFU/mL.	[55]
Alg + CS	*Bacillus coagulans* (BC)	Treatment of colitis and abdominal pain associated with irritable bowel syndrome.	Less than 1 log reduction in CFU was observed in SGF conditions and less than 2 log reduction in CFU was observed in 4% bile salts after 2 h. Adhesion of encapsulated BC is nearly 1.5 times higher than that of ordinary BC.	[56]
Alg + CS	Lactoferm ABY 6 ^1^	Prepare Greek yogurt.	The survival of bacteria in the simulated gastrointestinal environment was significantly improved.	[57]
Alg + CS	*Lactobacillus plantarum* KCC-42	Against different pathogenic fungal strains such as *A. fumigatus*, *A. clavatus.*	Higher viability rate (7.48 × 10^5^ CFU/mL) and tolerance to high acidic/pancreatin medium compared in mock gastrointestinal fluids.	[58]
Alg + CS	*Bifidobacterium longum* strain DD98	Regulating intestinal flora, increasing immune function, improving lipid metabolism.	Temperature and pH stability are significantly improved. Higher viability of encapsulated *B. longum* (reduced by 1.27 log CFU at 120 min at pH 2.5). Higher viability of encapsulated *B. longum* (decreased by 2.68 log CFU at 120 min when exposed to intestinal fluid with bile salt (1%)).	[59]
Alg + CS	*Lactobacillus gasseri* *Bifidobacterium bifidum*	Prevent gastrointestinal diseases.	Cell survival after exposure to SGF for 5 min was 95% of the initial population found in microencapsulated bacteria. Survival after incubating at SIF for 120 min, 98.86% and 96.72%.	[60]
Alg + CS + carboxymethyl cellulose (CMC)	*Bacillus subtilis natto*	Protect and promote the growth of probiotics.	Higher embedding rate (67.3%). Sustained release lasted for more than 10 h. Shelf life of viable *Bacillus subtilis natto* lasted for up to 20 days.	[61]
Alg + Hi maize + CS	*Lactobacillus acidophilus*	--	Longer survival at room temperature (6 months), freezing temperature (135 days), and cold storage temperature (105 days).	[62]
Alg + WPC ^2^/WPH ^3^	Lactoferm ABY 6	Prepare Greek yogurt.	More cells survived (more than 96%) after 4 h of gastrointestinal tract simulation compared with free culture cell (25.67%).	[63]
Alg + WPC/WPH	Lactoferm ABY 6	Prepare Greek yogurt.	High efficiency of encapsulation (between 92.98 and 94.20%).	[64]
Alg + WPI ^4^/DWPI ^5^	*Lactobacillus plantarum* (mtcc 5422)	Improve the intestinal microenvironment.	DWPI + Alg improved probiotics survival (96% by freeze-drying and 87% by spray-drying).	[65]
Alg + WPI	*Lactobacillus acidophilus*	--	By increasing the concentration of WPI, efficiency of encapsulation was significantly increased to 81.42–97.51%.	[66]
Alg + WSSM ^6^	*Lactobacillus casei*	--	The log reduction of encapsulated bacteria after 120 min incubation in SGF and SIF was 3.58–4.52 compared to 6.53 for free cells.	[67]
Alg + acidified egg albumen (EA) + stearic acid (SA) + cassava starch	*Lactobacillus acidophilus*	--	Less reduction of EA–SA-coated cells wrapped in microcapsules (1.3~0.6 log CFU/g) compared with free cells by exposure to moist heat at 70 °C for 30 min.	[68]
Alg + protamine	*Lactobacillus casei*	Improve intestinal flora, enhance immunity, inhibit tumor growth.	Survival 60 times higher in SGF compared to free cells. The speed of whole release process of encapsulated *L. casei* in pH 7.0 SIF is 7.6 times faster than the control group.	[69]

^1^ Lactoferm ABY 6 is lyophilized mixture of *Streptococcus salivarius* ssp. *thermophilus* (80%), *Lactobacillus acidophilus* (13%), *Bifidobacterium bifidum* (6%), *Lactobacillus delbrueckii* ssp. *bulgaricus* (1%). ^2^ WPC is short for “whey protein concentrate”. ^3^ WPH is short for “whey protein hydrolysate”. ^4^ WPI is short for “whey protein isolate”. ^5^ DWPI is short for “denatured whey protein isolate”. ^6^ Wild sage seed mucilage (WSSM) is a galactomannan with a rigid rod-like conformation.

**Table 3:** Microencapsulation of probiotics with physically modified alginate.

Coating Material	Encapsulated Strain	Application	Remarks	References
Alg + cassava starch	*Rhodopseudomonas palustris* KTSSR54	Promote plant growth.	The encapsulation efficiency with alginate alone was 50.56%, compared to 70.83% when the starch content was 4% (*w*/*v*).	[99]
Alg + corn starch	*Lactobacillus fermentum* CECT5716	--	Encapsulation efficiency of starch particles increased significantly (from 74.41% to 97.26%).	[100]
Alg + native corn starch	*Lactobacillus casei* 01	--	Beads with a starch concentration of 600 g/L had 4 times more storage stability than without starch and had a porosity rate of 1/3. After lyophilization, the survival of cells encapsulated within the beads with starch was 100 times higher than that of the control group.	[101]
Alg + corn starch	*Lactobacillus acidophilus* (LA5)*, Lactobacillus rhamnosus* 23,527 LGG, *Bifidobacterium bifidum, Bifidobacterium animalis*	--	The viability rate of lactobacilli and bifidobacteria, after 120 min, in gastric condition obtained 90% and 84.1% and in intestinal condition 71.7% and 77.8% of the initial count compared to encapsulated control, respectively, (*p* < 0.01).	[102]
Alg + corn starch	*Lactobacillus rhamnosus* 23,527 LGG, *Lactobacillus acidophilus* (LA5), *Bifidobacterium bifidum, Bifidobacterium animalis*	--	Temperature stability is significantly improved. The survival rates of nanoencapsulated LAB and *Bifidobacterium* increased from 87.7% to 97.9% and from 86.3% to 96.9%, respectively, after 20 days of yogurt preservation.	[103]
Alg + Hi-maize	*Lactobacillus acidophilus*	--	The freeze-dried microparticles of alginate and alginate + Hi-maize had diameters of 114.51 and 78.49 µm, respectively.	[104]
Alg + carboxymethylpachymaran (CMP)	*Lactobacillus plantarum*	--	The composite coating showed excellent performance in terms of sustainable release and freeze-drying stability: 10^7^ CFU/mL before and after freeze-drying. Composite coating has good storage stability: 2.09 × 10^6^ CFU/mL at 4 °C for 90 days.	[105]
Alg + fish gelatin	*Lactobacillus acidophilus* LA-5	--	Compared with the control (free bacteria), the activity of encapsulated *L*. *acidophilus* increased to 2.49 and 3.07 log CFU/g during baking and storage, respectively. Good storage stability: 10^6^ CFU/g after 4 days.	[106]
Alg + polystyrene (PS)	*Lactobacillus plantarum* 2675	--	Temperature and UV light had low inhibitory effects on the growth of immobilized cells, from 81% to 40% and from 64% to 48%, respectively.	[107]
Alg + Poly(vinyl alcohol)(PVA)	*Lactobacillus* *rhamnosus GG*	Treatment of allergic diseases	Zeta potential values for VS ^1^ and VSPBe ^2^ were −6.29 mV and −7.74 mV, respectively. The bacterial survival rate was up to 9.62 log CFU/mL 11% (1st day), 37% (3rd day), 29% (5th day), and 8% (9th day) decrement in TMAB ^3^ counts of coated fish fillets compared to uncoated fish fillets (*p* < 0.05).	[108]
Alg + PVA	*Lactobacillus paracasei* KS-199	--	Enhanced protection ability of the mats was observed in thermal degradation assays (weight loss from 93.4 to 84.5%). Increased survival of strains in simulated gastric juice (the viability rate from 64.1 to 70.8 log CFU/mL).	[109]

^1^ VS is short for “poly (vinyl alcohol) & sodium alginate-based nanofibers”. ^2^ VSPBe is short for “probiotic bacteria-loaded poly (vinyl alcohol) & sodium alginate-based nanofibers”.^3^ TMAB is short for “total mesophilic aerobic bacteria”.

## Data Availability

Not applicable.
